# Revisiting Eosinophilia: A Neglected Indicator of Adrenal Insufficiency

**DOI:** 10.7759/cureus.80988

**Published:** 2025-03-22

**Authors:** Thi My Nguyet Nguyen, Quynh Nguyen, Hoang Dinh, Van Phan, Vien X Phan

**Affiliations:** 1 Internal Medicine, Interfaith Medical Center, One Brooklyn Health, New York City, USA; 2 Internal Medicine, Logan Regional Medical Center, West Virginia, USA; 3 Internal Medicine, Ho Chi Minh City Medicine and Pharmacy University, Ho Chi Minh, VNM; 4 Endocrinology, Diabetes and Metabolism, Middlesex Hospital, Connecticut, USA

**Keywords:** adrenal insufficiency, covid-19 vaccine, eosinophilia, hyponatremia, hypothyroidism

## Abstract

Diagnosing adrenal insufficiency is challenging due to its nonspecific symptoms and insidious onset. We report the case of a 55-year-old woman who developed persistent eosinophilia accompanied by nausea, vomiting, fatigue, muscle weakness, dizziness, and brain fog following her second COVID-19 vaccination. A comprehensive evaluation ultimately led to the diagnosis of adrenal insufficiency. Her symptoms and eosinophilia resolved with corticosteroid therapy.

This case demonstrated an unusual presentation of adrenal insufficiency and the importance of considering it in the differential diagnosis when eosinophilia is present. By remaining vigilant regarding the diverse clinical presentations of adrenal insufficiency, we can facilitate timely diagnosis with a more cost-effective approach.

## Introduction

Adrenal insufficiency is a rare but potentially life-threatening endocrine disorder characterized by inadequate glucocorticoid production, often with mineralocorticoid deficiency due to dysfunction at the adrenal gland, pituitary, or hypothalamus level. Common causes include autoimmune destruction (Addison’s disease), long-term steroid use, pituitary tumors, infections like tuberculosis, and sudden withdrawal of corticosteroids. Diagnosing adrenal insufficiency can be challenging due to its nonspecific symptoms, such as nausea, fatigue, muscle weakness, and cognitive impairment, which overlap with numerous other conditions.

Here, we present a case of a 55-year-old woman who developed persistent eosinophilia following her second COVID-19 vaccination, accompanied by systemic symptoms suggestive of adrenal insufficiency. Her eosinophilia and symptoms improved with steroid treatment. This case highlights the importance of considering adrenal insufficiency in the differential diagnosis of eosinophilia, reinforcing the need for clinical vigilance in recognizing atypical presentations.

This article was previously presented as a poster abstract at the Endocrine Society ENDO 2024 on June 2, 2024.

## Case presentation

**Figure 1:**
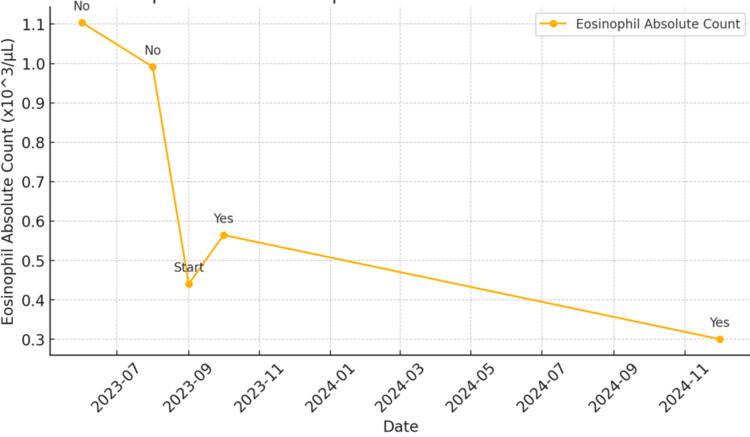
Relationship between eosinophil absolute count and steroid treatment

**Figure 2:**
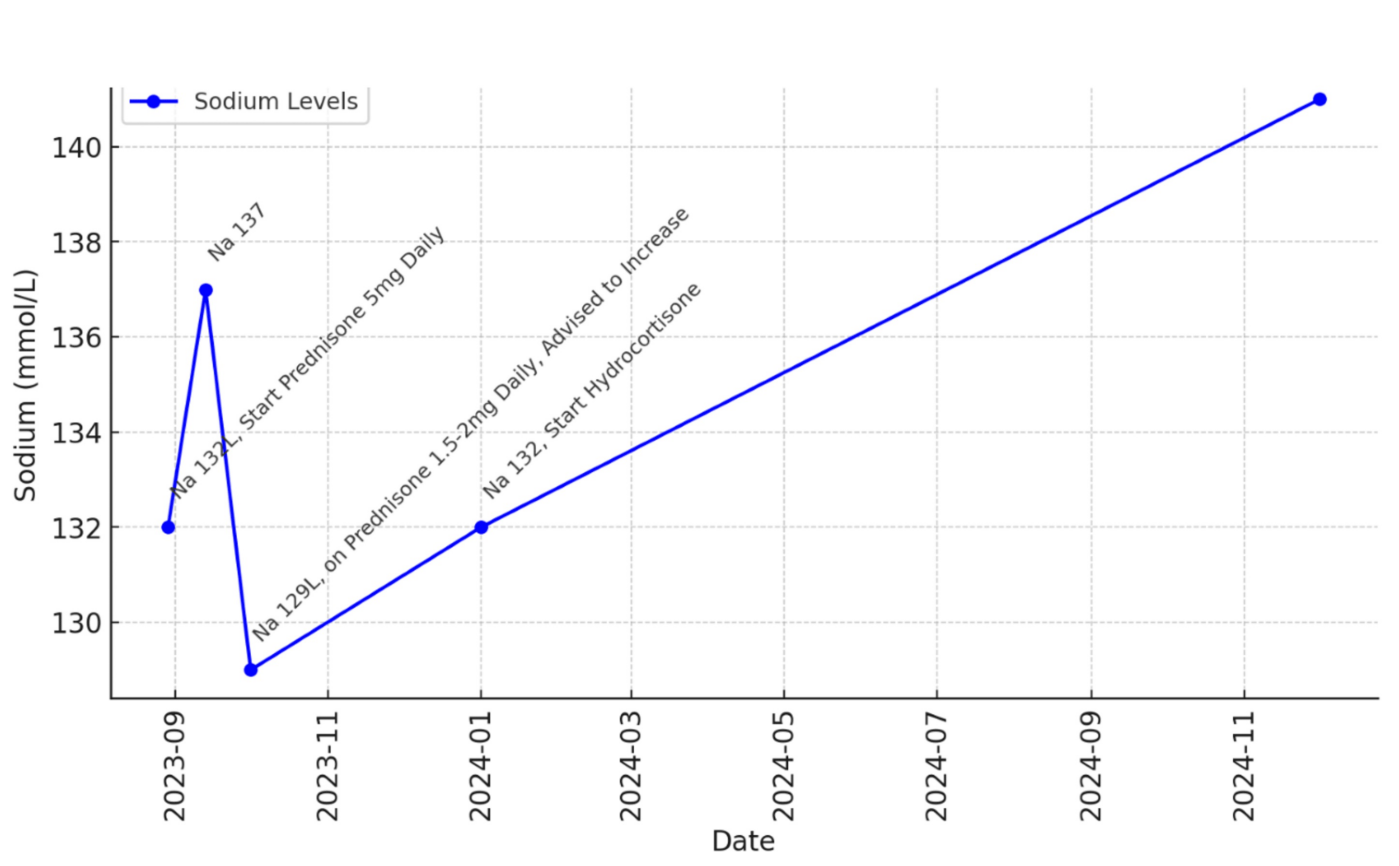
Relationship between sodium level and steroid treatment

A 55-year-old menopausal woman with an unremarkable medical history, except for a 2015 episode of fever of unknown origin, and no known allergy. Following her second COVID-19 vaccination in 2021, she developed persistent fatigue. Since then, her CBC has consistently shown eosinophilia. An extensive workup, including a bone marrow biopsy, was conducted to rule out allergic conditions, parasitic infections, autoimmune diseases, and neoplastic processes, but the results were inconclusive. A late allergic reaction to the COVID-19 vaccine was considered, leading to the initiation of empirical prednisone 10mg daily with a gradual tapering of the prednisone over about a month. Her symptoms improved and eosinophil normalized during the treatment course. However, eosinophilia recurred once the patient discontinued prednisone treatment. Subsequent blood tests in June 2023 and August 2023 showed eosinophil counts of 23% (normal range 0%-5%) with absolute eosinophil count of 1,120 cells/µL (normal range 0-500 cells/µL) and 13% (absolute eosinophil count 0.5), respectively. Her comprehensive metabolic panel also revealed hyponatremia with a serum sodium level of 126 mmol/L (normal range 135-145 mmol/L), which led to an evaluation of thyroid function, which indicated primary hypothyroidism. Despite three months of levothyroxine replacement, which appropriately decreased TSH levels, hyponatremia persisted, raising concerns for other additional etiologies. The patient also experienced nausea, vomiting, generalized weakness, dizziness, joint pain, and brain fog. Further workup showed extremely low random cortisol level of 0.7 mcg/dL, raising concern for adrenal insufficiency. This was confirmed by inadequate response to the corticotropin stimulation test. Baseline serum cortisol was 0.7 mcg/dL. Cortisol levels at 30 and 60 minutes post-injection were 3.9 and 5.5 mcg/dL, respectively. Adrenal insufficiency was confirmed. Consequently, prednisone 5 mg daily was initiated. On examination, the patient appeared comfortable, with a blood pressure of 99/60, and heart rate of 73. There were no signs of cutaneous or mucosal hyperpigmentation, vitiligo, Cushingoid appearance, or thyroid enlargement. The patient reported improvement in her energy and muscle strength. Her hyponatremia resolved and the eosinophil count normalized after two weeks of steroid use. Therefore, hyponatremia and eosinophilia were likely a result of adrenal insufficiency (Figures 1, 2).

Lab results were collected when the patient was at our clinic before taking her daily prednisone dose (Table 1).

**Table 1:** Lab results

Test	Result	Normal range
Cortisol (morning)	5.5 µg/dL	6 - 23 µg/dL
Adrenocorticotropic hormone (ACTH)	12 pg/mL	10 - 60 pg/mL
Sodium (Na)	137 mmol/L	135 - 145 mmol/L
Potassium (K)	4.2 mmol/L	3.5 - 5.0 mmol/L
Renin	0.41 ng/mL	0.2 - 1.0 ng/mL/hr
Aldosterone	4 ng/dL	4 - 31 ng/dL
21-hydroxylase antibodies	Negative	Negative
Thyroid-stimulating hormone (TSH)	3.011 mU/L	0.4 - 4.0 mU/L
Free T4	1.4 ng/dL	0.7 - 1.9 ng/dL
Triiodothyronine (T3)	79 ng/dL	70 - 204 ng/dL
Thyroglobulin antibodies (TgAb)	Negative	Negative
Thyroid peroxidase antibodies (TPOAb)	Negative	Negative
Follicle-stimulating hormone (FSH)	132 mIU/mL	3 - 20 mIU/mL (varies with cycle)
Luteinizing hormone (LH)	53 mIU/mL	2 - 15 mIU/mL (varies with cycle)
Prolactin (Prl)	10.7 ng/mL	4.8 - 23.3 ng/mL
Insulin-like growth factor 1 (IGF-1)	89 ng/mL	45 - 200 ng/mL

Hydrocortisone was prescribed as a replacement for prednisone; however, the patient was unable to tolerate it due to gastrointestinal discomfort and subsequently reverted to prednisone at a dose of 5 mg daily. Due to complaints of insomnia, the patient independently reduced the prednisone dose gradually to 1.5-2 mg per day. This dose reduction led to the recurrence of hyponatremia and hypereosinophilia, along with persistent fatigue. Consequently, prednisone was discontinued, and hydrocortisone was reintroduced, which the patient is now tolerating well. Follow-up clinic visits were scheduled for medication adjustment and further evaluation, including a brain MRI, but the patient declined due to her personal preference. 

## Discussion

Peripheral blood eosinophilia is defined as an absolute eosinophil count ≥500 eosinophils/µL and may be caused by numerous conditions, including allergic, infectious (especially tissue-invasive parasites), inflammatory, autoimmune disorders, neoplastic disorders, and metabolic conditions like adrenal insufficiency [1]. The association between eosinophilia and adrenal insufficiency, particularly in cases of Addison's disease, was first described by George W. Thorn in the mid-20th century. He noted that when cortisol levels were low (as in adrenal insufficiency), the body was less able to suppress eosinophil production, leading to the characteristic rise in eosinophils seen in these patients [2]. While Thorn's work was a significant step in identifying this relationship, much of the research since then has continued to confirm the link between eosinophilia and adrenal insufficiency, with the understanding that eosinophil counts can serve as an indirect marker of cortisol deficiency. However, the mechanism remains complex and multifactorial. To date, it is known that cortisol plays a role in suppressing eosinophil counts by promoting eosinophil apoptosis, reducing bone marrow eosinophil production, as well as shifting eosinophils from blood vessels into tissue space [3]. Studies have shown that corticosteroid administration rapidly reduces eosinophil counts in patients with Addison’s disease. For example, a case report of a 21-year-old male with untreated Addison’s disease documented a 70% reduction in eosinophil counts within four hours of corticosteroid administration. Bone marrow studies further demonstrated a significant decline in eosinophil production and proliferation, reinforcing that eosinophilia in adrenal insufficiency stems from cortisol deficiency rather than an intrinsic hematologic disorder [4]. Additionally, a prospective study in critically ill patients has suggested that an increase in circulating eosinophils may serve as an indicator of relative adrenal insufficiency in this population [5].

In our case, persistent eosinophilia resolved during empirical prednisone therapy but recurred upon discontinuation, mirroring the well-documented cortisol-eosinophil relationship. This pattern, alongside laboratory findings of low cortisol levels and an inadequate adrenocorticotropic hormone (ACTH) stimulation test response, strongly suggests that eosinophilia served as an early clinical clue to underlying adrenal insufficiency. The coexistence of hyponatremia further supports this diagnosis, as cortisol deficiency impairs free water excretion, leading to dilutional hyponatremia - a well-recognized feature of adrenal insufficiency. A notable limitation in this case is the absence of a baseline ACTH level before initiating corticosteroid therapy, which makes it difficult to distinguish between primary and secondary adrenal insufficiency. Although the patient’s ACTH and renin levels were within the normal range while on prednisone, chronic steroid use may have suppressed endogenous ACTH production, masking the underlying pathology. Additionally, the negative 21-hydroxylase antibody result does not definitively rule out autoimmune adrenal insufficiency, as seronegative Addison’s disease has been documented in some cases. Given the patient’s concurrent primary hypothyroidism, most likely due to Hashimoto’s thyroiditis despite negative thyroid autoantibodies, the possibility of autoimmune polyglandular syndrome (APS) should be considered. APS type 2 is characterized by the coexistence of primary adrenal insufficiency, autoimmune thyroid disease, and, in some cases, type 1 diabetes mellitus. However, we are unable to complete the investigations because the patient refused further evaluation.

This case underscores the importance of recognizing eosinophilia as a potential early marker of adrenal insufficiency, a relationship that has been described in the literature but remains underutilized in clinical practice. By maintaining a high index of suspicion, clinicians may facilitate earlier diagnosis and avoid unnecessary diagnostic delays. Future research should explore the role of eosinophilia as a cost-effective and accessible screening tool for adrenal insufficiency, particularly in patients presenting with unexplained persistent eosinophilia.

## Conclusions

This case highlights an uncommon presentation of adrenal insufficiency and the importance of considering it in the differential diagnosis when eosinophilia is present. The association between eosinophilia and adrenal insufficiency is often overlooked, leading to potential delays in diagnosis. Increased awareness of this relationship can aid in earlier recognition, allowing for timely treatment and improved patient outcomes.
